# Circulating Tumor Necrosis Factor Alpha May Modulate the Short-Term Detraining Induced Muscle Mass Loss Following Prolonged Resistance Training

**DOI:** 10.3389/fphys.2019.00527

**Published:** 2019-05-03

**Authors:** Gerard McMahon, Christopher I. Morse, Keith Winwood, Adrian Burden, Gladys L. Onambélé

**Affiliations:** ^1^Sport and Exercise Sciences Research Institute, Ulster University, Belfast, United Kingdom; ^2^Musculoskeletal Science and Sports Medicine Research Centre, Manchester Metropolitan University, Crewe, United Kingdom

**Keywords:** cytokine, inflammation, muscle architecture, specific force, young

## Abstract

**Introduction:**

Tumor necrosis factor alpha (TNFα) is a pro-inflammatory cytokine that has been shown to modulate muscle mass, and is responsive to exercise training. The effects of resistance training (RT) followed by a short period of detraining on muscle size, architecture and function in combination with circulating TNFα levels have not been previously investigated in a young, healthy population.

**Methods:**

Sixteen participants (8 males and 8 females) were randomly assigned to a training group (TRA; age 20 ± 3 years, mass 76 ± 7 kg), whilst fourteen participants (7 males and 7 females) age 22 ± 2 years, mass 77 ± 6 kg were assigned to a control group (CON). Measures of vastus lateralis (VL) muscle size (normalized physiological cross-sectional area allometrically scaled to body mass; npCSA), architecture (fascicle length; L_F_, pennation angle Pθ), strength (knee extensor maximal voluntary contraction; KE MVC), specific force, subcutaneous fat (SF) and circulating TNFα were assessed at baseline (BL), post 8 weeks RT (PT), and at two (DT1) and four (DT2) weeks of detraining.

**Results:**

Pooled BL TNFα was 0.87 ± 0.28 pg/mL with no differences between groups. BL TNFα tended to be correlated with npCSA (*p* = 0.055) and KEMVC (*p* = 0.085) but not specific force (*p* = 0.671) or SF (*p* = 0.995). There were significant (*p* < 0.05) increases in npCSA compared to BL and CON in TRA at PT, DT1, and DT2, despite significant (*p* < 0.05) decreases in npCSA compared to PT at DT1 and DT2. There were significant (*p* < 0.05) increases in L_F_, Pθ and KE MVC at PT but only L_F_ and torque at DT1. There were no significant (*p* > 0.05) changes in SF, specific force or TNFα at any time points. There was a significant correlation (*p* = 0.022, *r* = 0.57) between the relative changes in TNFα and npCSA at DT2 compared to PT.

**Discussion:**

Neither RT nor a period of short term detraining altered the quality of muscle (i.e., specific force) despite changes in morphology and function. TNFα does not appear to have any impact on RT-induced gains in muscle size or function, however, TNFα may play a role in inflammatory-status mediated muscle mass loss during subsequent detraining in healthy adults.

## Introduction

Reduced levels of skeletal muscle mass and strength are associated with many chronic diseases and increased mortality in humans (Rantanen, 2003; Oterdoom et al., 2009; Kalyani et al., 2014; Bowen et al., 2015), understanding the fundamental adaptation of skeletal muscle is therefore, key to optimizing health and longevity. Skeletal muscle displays remarkable plasticity with its ability to alter its phenotype in response to mechanical/metabolic stimuli or lack thereof, with an increase (hypertrophy) or decrease (atrophy) in muscle fiber size, respectively (Coffey and Hawley, 2007; Wackerhage et al., 2018). Resistance exercise is well established as a potent stimulus for acutely altering muscle protein synthetic rates (MacDougall et al., 1995), and following repeated resistance training (RT) sessions over a period of time, this stimulus ultimately results in an increase in skeletal muscle mass and strength [e.g., (Moritani and DeVries, 1979; Young et al., 1983; Frontera et al., 1988; McMahon et al., 2012, 2014, 2018)]. During a period of detraining (DT) however, preceding gains in muscle fiber size are rapidly diminished. For example, in a mouse model, Jespersen et al. (2011), demonstrated that following 90 days of RT, almost 50% of the RT-induced increase in muscle fiber size was lost after only 10 days of DT. In humans, detraining in the form of 5–6 weeks immobilization, following 5–6 months of RT, resulted in a 41% loss of RT-induced gains in elbow flexor strength, and 33 and 25% reduction in fast/slow twitch muscle fiber size, respectively, in healthy male subjects (MacDougall et al., 1980). Early work from Häkkinen and Komi (1983) demonstrated that following 16 weeks of strength training in young, healthy males, muscle strength and integrated electromyography (iEMG) of the leg extensors were significantly increased. Following a subsequent 8 week detraining period, the significant reductions in muscle force were accounted for by a reversal of the preceding RT induced gains in neuromuscular variables. More recently, Blazevich et al. (2007) reported that following 10 weeks of either concentric or eccentric RT in young men and women, there were significant improvements in muscle volume, physiological cross-sectional area (pCSA), architecture and training mode specific strength. Following 3 months of detraining, there was a significant, contraction mode specific, decrement in strength (eccentric only) and interestingly non-significant changes in hypertrophic and architectural measures. Despite this evidence in RT and subsequent DT studies of longer durations, which may reflect different patterns of adaptation to RT and DT, periods of DT following RT and the associated alterations to muscle mass and function to date have been poorly characterized in young, healthy adults following short-term detraining such as 1–4 weeks’ post-RT. To the authors’ knowledge, there are only a few studies that have been published previously that have reported changes to muscle mass and strength following short-term DT (≤4 weeks) following a RT only program (i.e., not part of a concurrent RT and endurance training program) (Kubo et al., 2010; McMahon et al., 2012; Yasuda et al., 2015). However, large methodological discrepancies make these studies incomparable due to factors such as muscle groups analyzed, length of the preceding training period, measurement points during detraining, and differences in muscle size and strength measurements. Our paper has previously demonstrated that muscle size (anatomical cross-sectional area [aCSA]) and maximal voluntary contraction (MVC) torque is significantly elevated above baseline following 8 weeks of RT and remained elevated following a further 2 and 4 weeks of detraining (McMahon et al., 2012), although these varied slightly depending on the training group. Two factors that are critical in fully describing muscles’ characteristics are physiological cross-sectional area (the area of muscle at right angles to the longitudinal axis of the fibers) and the muscle’s specific force (normalization of force per unit pCSA) which, in combination with neural factors (Moritani and DeVries, 1979; Del Vecchio et al., 2019) provide a true representation of the muscle size and intrinsic force generating capacity. Neither pCSA nor specific force have been investigated in short-term (≤4 weeks) detraining studies, with specific force, to the author’s knowledge, not within any length of detraining studies following a period of RT.

In addition to RT acting as stimuli for phenotype changes, the endocrine system also plays a critical role in phenotype expression by having the ability to tip the balance of muscle cellular anabolism and catabolism through influence of various growth factors and cytokines (Solomon and Bouloux, 2006). One such cytokine (or myokine when secreted from muscle) that plays an integral role in altering muscle state is tumor necrosis factor alpha (TNFα). TNFα is a pro-inflammatory cytokine that is associated with the modulation of muscle tissue loss, particularly in pathological disease such as cancer cachexia (Balkwill, 2006) and Sarcopenia (Visser et al., 2002). For example, high levels of circulating IL-6 and TNFα have been associated with lower muscle mass and strength in well-functioning older adults (Visser et al., 2002). Loss of specific muscle proteins such as myosin heavy chain (MHC) content are modulated by TNFα- mediated effects on the p38 MAPK and nuclear factor-κB (NF-κB) pathways of the ubiquitin-proteasome pathway (Li et al., 1998, 2005; Li and Reid, 2000). Li et al. (1998) demonstrated that following 72 h of 1–6 ng/mL of TNFα treatment on differentiated mouse skeletal muscle C2C12 myotubes, there were significant protein content losses. Furthermore, concentrations as low as 1–3 ng/mL induced losses of adult MHC protein in a concentration dependent manner in primary cultures from rat skeletal muscle. These losses were not associated with a decrease in muscle DNA content or a decrease in MHC protein synthesis, which is reflective of chronic muscle wasting (Li et al., 1998). In a subsequent study from the same group (Li et al., 2005) also demonstrated that following intraperitoneal injection of 6 ng/mL of TNFα, Atrogin-1/MAFBx expression increased by 76.2 ± 17.1% after 4 h and 87.7 ± 10.1% after 6 h in adult gastrocnemius muscles of mice. Previous research into the impact of RT on TNFα levels has been mainly conducted in only elderly or diseased populations, with only sparse data in a young population currently available. Other methodological issues within the literature is that many studies have investigated concurrent training (i.e., combinations of RT and endurance training), not singularly RT and provide little or no insight into changes in muscle mass or function. Louis et al. (2007) reported that following an acute bout of RT in young adults, TNFα mRNA was significantly increased immediately and for up to 24-h post-RT. In a study by Townsend et al. (2013) also investigating the acute response to RT, the authors showed that in resistance-trained men, circulating TNFα was significantly increased immediately post-RT compared to pre-RT. However, TNFα levels had returned to baseline within 30 min post-RT, remaining as such for a further 24 and 48 h. This is in somewhat of agreement with the study of Alves et al. (2013) who demonstrated that following either eccentric or concentric RT only, there were no acute increases in TNFα post-RT (24–96 h). Furthermore, this study also showed that following 10 RT sessions over 3 weeks, there were no significant changes in circulating TNFα 24–96 h post the final RT session. Paoli et al. (2015) report that following 8 weeks of upper body RT in combination with either a high-or-low protein diet, despite no significant alterations in the acute circulating TNFα response, the authors noted chronic RT could elevate both the basal and RT-induced response of circulating TNFα. The authors of this previous study, however, report neither changes in muscle mass or strength. Furthermore, data from Bruunsgaard et al. (2004) demonstrate that muscle strength after 12 weeks of RT in 86–95 year olds is inversely correlated with TNFα receptor content, showing that the TNFα system can also impact on muscle function. Currently there are no investigations on TNFα levels and concomitant changes in muscle size and strength in younger adults following a sustained RT program only, and following a period of short-term detraining.

Therefore, the aims of this study were to (1) investigate the effects of RT and DT on muscle size and function, (2) describe changes in TNFα levels following RT and DT, and (3) probe whether there is any link between changes in muscle size and function and circulating TNFα levels in a young, healthy population.

## Materials and Methods

### Participants

Thirty young participants recruited from the local university campus, gave written informed consent to participate in the study in accordance with the Declaration of Helsinki. All procedures and experimental protocols were approved by the Manchester Metropolitan University Cheshire Campus ethics committee. Exclusion criteria included the presence of any known musculoskeletal, neurological, inflammatory, or metabolic disorders or injury. Participants took part in recreational activities such as team sports and had either never taken part in lower limb RT or had not done so within the previous 12 months. Each participant completed a physical activity diary, outlining that they each habitually completed 3–5 h of non-resistance based moderate physical activity per week. Sixteen participants were randomly assigned to a training group (TRA) age 20 ± 3 years, mass 76 ± 7 kg), whilst fourteen participants (7 males and 7 females) age 22 ± 2 years, mass 77 ± 6 kg were assigned to a control group (CON).

### Study Design

The study design was convenience sampling, with participants separated into groups by random allocation to one of two groups (i.e., training or control). Following familiarization with laboratory procedures at least 1 week prior to testing proper, participants were assessed for muscle morphology, architectural and functional properties and TNFα at baseline (week 0). Measurements were repeated after 8 weeks of RT (post-training [PT]), week 10 following 2 weeks of detraining (DT1) and week 12 following a further 2 weeks detraining (DT2).

### Resistance Training

Resistance training was performed three times per week for 8 weeks at 80% of 1 repetition maximum (1RM) on the knee extensor complex. Exercises included the back squat, leg press, leg extension (Technogym, Berkshire, United Kingdom), lunge, Bulgarian split squat and Sampson chair. All exercise sessions were supervised by a member of the research team. Participants completed two familiarization sessions at 70%1RM prior to commencing the RT program. 1RMs were measured at baseline and every 2 weeks, with loading weight progressed. Each training session consisted of four exercises with participants performing three sets of 10 repetitions per exercise for the first 4 weeks, and four sets of eight repetitions per exercise thereafter. Training records were diligently completed during sessions. Following RT, participants returned to habitual daily activities with no form of exercise training permitted for the detraining period (4 weeks).

### Assessment of Muscle Morphology

All muscle morphological and architectural measures were taken at rest with each participant seated in an upright position on an isokinetic dynamometer (Cybex, CSMi, MA, United States). After calibration, each participant was positioned with a hip angle of 80° (straight back 90°) and knee at 90° knee flexion (straight leg 0°). All measurements were determined using ultrasonography (AU5, Esaote Biomedica, Genoa, Italy) at rest with a 40 mm probe, with images captured at 25 Hz using a digital video recorder (Tevion, Medion Australia Pty Ltd., St Leonards, Australia). The measurement sites were 25, 50, and 75% of femur length. Femur length was defined as the line passing from the greater trochanter to the central palpable point of the space between the femur and tibia heads when the knee was flexed at 90°. Vastus lateralis fascicle pennation angle (θ) was measured as the angle of fascicle insertion into the deep aponeurosis. Images were obtained perpendicular to the dermal surface of the VL and oriented along the midsagittal plane of the muscle. The transducer was then aligned in the fascicle plane to capture an optimal number of clearly demarked fascicles. Images were taken at 25, 50, and 75% of the total femur length (as described below) and 50% of muscle width at each point (where 50% muscle width is defined as the midpoint between the fascia separating the VL and rectus femoris, and fascia separating the VL and biceps femoris muscles). Fascicle length was defined as the length of the fascicular path between the deep aponeurosis and superficial aponeurosis of the VL. The majority of fascicles extended off the acquired image, where the missing portion was estimated by linear extrapolation. This was achieved by measuring the linear distance from the identifiable end of a fascicle to the intersection of a line drawn from the fascicle and a line drawn from the superficial aponeurosis. This method has been shown to produce reliable results previously (Blazevich et al., 2007). All images were analyzed and measured using Image J v.1.43c (National Institutes of Health, Bethesda, MD, United States). Subcutaneous fat was estimated using the same images as taken for muscle architecture and analyzed at 50% femur length only. After calibration in Image J to coincide with the scale of the ultrasound image, a line from the top to the bottom of the layer of fat visualized was drawn at three regular intervals on the ultrasound image. The average lengths of these three lines were taken to estimate the average thickness of the subcutaneous fat layer in millimeters. Care was taken not to deform or compress the subcutaneous fat with minimal pressure applied to the dermal surface with the ultrasound probe.

For a full description of aCSA methods please see Reeves et al. (2004a) The aCSA was measured with the probe aligned in the axial plane. Echo-absorptive tape was placed at regular intervals (∼3 cm) along the muscle width at each site so that when the probe was placed on the leg, two distinct shadows were cast on the ultrasound image. Therefore, each ultrasound image provided a section of VL within the boundaries set by the two shadows and fascia surrounding the muscle. Each of these sections was analyzed for the total area using Image J to provide a total aCSA at that particular site.

The measurement techniques used for the calculation of physiological cross-sectional area of the vastus lateralis (VL) muscle in the current study has been previously documented elsewhere (Reeves et al., 2004a; McMahon et al., 2014). Using the aCSA measures, muscle volume was then calculated using the truncated cone method, which has been validated in a number of previous studies (Esformes et al., 2002; Reeves et al., 2004b). VL pCSA was calculated by dividing muscle volume by fascicle length (Reeves et al., 2004b). Allometric scaling was then used to normalize (npCSA) to body mass (Jaric et al., 2005).

### Knee Extensor Maximal Voluntary Contraction (KE MVC) and Vastus Lateralis Specific Force

Prior to this assessment, a number of measures were taken to minimize inaccuracies during the dynamometer assessments, thereby counteracting any potential effect of soft tissue compliance, dynamometer alignment, as well as gravitational forces. With these precautions in place, maximal isometric knee extension torque was measured with the knee at 70° knee flexion (full knee extension = 0°) on the right leg of all participants, corresponding to the angle of peak torque. After a series of warm up trials consisting of ten isokinetic contractions at 60°.s^−1^ at self-perceived 50–85% maximal effort, participants were instructed to rapidly exert maximal knee extensor isometric force (maximal voluntary contraction, KE MVC) against the dynamometer lever arm. Joint torque data traces were displayed on the screen of a MacBook Air computer (Apple Computer, Cupertino, CA, United States), which was interfaced with an A/D system (Acknowledge, Biopac Systems, Santa Barbara, CA, United States) with a sampling frequency of 2000 Hz. Isometric contractions were held for ∼2–3 s at the plateau with a 60 s rest period between contractions. Peak torque was expressed as the average of data points over a 500 ms period at the plateau phase (i.e., 250 ms either side of the instantaneous peak torque). The peak torque of three extensions was used as the measure of torque in each participant.

A pair of Ag-AgCl electrodes (Neuroline 720, Ambu, Denmark), were placed on clean, shaved, and abraded skin, at 50% of femur length, in the mid-sagittal plane of the biceps femoris. The reference electrode (Blue sensor L, Ambu, Denmark) was placed on the lateral tibial condyle. The raw EMG signal was preamplified (MP100, Biopac Systems Inc., United States), amplified × 1000 (MP100, Biopac Systems Inc., United States), bandpass filtered between 10 and 500 Hz (Biopac Systems, United States) with a notch at 50 Hz, and sampled at 2000 Hz. All EMG and torque signals were displayed in real time in AcqKnowledge software (Biopac systems Inc., United States) via a PC. Two maximal knee flexion contractions were carried out to obtain the EMG at maximal flexion torque. The root mean square (RMS) EMG activity was averaged for a 500 ms period (average of 1.5 ms moving windows) which coincided with the plateau of peak KE MVC torque. To reiterate, the EMG of the long head of the biceps femoris muscle was measured to ascertain the level of antagonist muscle co-contraction during knee extensor MVC. The biceps femoris torque during a knee-flexion contraction was calculated as described by McMahon et al. (McMahon et al., 2014) whereby a linear relationship between BF EMG and KE MVC torque is assumed, thus enabling the quantification of the “pull back torque” during knee extensions, and ultimately, the total forces experienced by the patella tendon (Pearson and Onambele, 2005, 2006).

Muscle specific force was calculated in multiple steps as previously reported (Reeves et al., 2004b). Briefly, VL fascicle force was calculated by dividing the estimated VL muscle force by the Cosθ angle of pennation at 50% femur length. Specific force of the VL muscle was calculated at the knee joint angle where maximal fascicle force peaked, which corresponded to the knee joint angle of 70° before and after training. Specific force was calculated by dividing fascicle force by pCSA.

### Circulating Tumor Necrosis Factor Alpha (TNFα) Levels

Pre-, post-training and during detraining, following an overnight fast, (∼10 h), participants reported to the laboratory between 9 and 11am. 5 mL blood samples were collected from the antecubital vein of the forearm, placed in a crushed ice bed for 1.5 to 2-h, and then centrifuged at 4°C for 10 min at 4,800 rpm, with the supernatant being removed and stored in at least two aliquots in eppendorf at −20° Celsius for later analysis. TNFα was analyzed using the standard enzyme-linked immuno-sorbent assay (ELISA) procedure, as described previously (McMahon et al., 2013). Post-training samples were taken 3–4 days post final training session, at the same time-of-day as the pre-training sampling for each participant, with detraining samples being taken twice fortnightly after the post-training sample date. The laboratory tests were timed to avoid diurnal variability or acute exercise-induced cytokine fluctuations. In CON group, TNFα analysis, *n* = 6 (3 males, 3 females) with TRG *n* = 16.

### Statistical Analyses

Data were parametric (as determined through Shapiro Wilk and Levene’s tests) and were therefore analyzed using a mixed-design repeated measures ANOVA. The within factor was the phase of training (i.e., weeks 0, 8, 10, and 12) and the between factor was group (TRG or CON). All data are presented as mean ± SD. Statistical significance was set with alpha at *p* ≤ 0.05. In terms of the sample size in this study, the average statistical power of the measured muscle parameters (VLCSA, pennation angle, fascicle length, and KE MVC was statistically adequate at beta = 0.89.

## Results

There were no significant differences (*p* > 0.05) between TRA and CON groups (independent *t*-tests) in any of the measured muscle variables, at baseline. Pooled baseline TNFα was 0.87 ± 0.28 pg/mL with no differences between groups. There was a tendency for baseline TNFα to be correlated with npCSA (*p* = 0.055) and also KEMVC (*p* = 0.085), however, there was no correlation with specific force (*p* = 0.671) or SF (*p* = 0.995).

### Muscle Morphology

**FIGURE 1:**
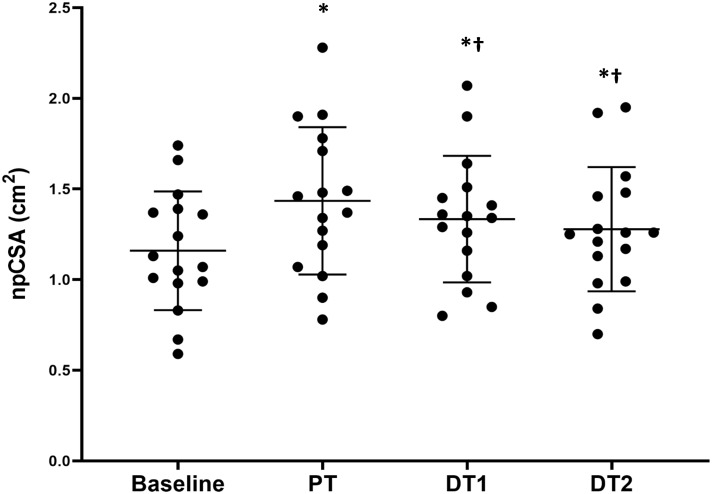
Changes in vastus lateralis normalized physiological cross-sectional area (npCSA) in TRA group at baseline, post-training (PT), detraining 1 (DT1), and detraining 2 (DT2). ^∗^Significantly different to baseline *p* < 0.001, ^†^Significantly different to PT *p* < 0.05.

There were significant absolute increases (*p* < 0.01) in npCSA compared to baseline at all time points (PT, DT1, and DT2) in TRA (Figure 1), with no changes in CON (*p* > 0.05). There were also significant relative changes in npCSA at all time points (26 ± 23%, 18 ± 20%, and 12 ± 19% for PT, DT1, and DT2, respectively) compared to baseline and controls (*p* < 0.05). When compared to PT, there were significant absolute (Figure 1) and relative (−6 ± 8% and −10 ± 8%) reductions in npCSA in TRA at DT1 and DT2, respectively, and versus controls (*p* < 0.05).

**Table 1:** Functional and architectural properties.

Variable	Absolute				Relative				

	BL	PT	DT1	DT2	PT vs. BL %	DT1 vs. BL %	DT2 vs. BL %	DT1 vs. PT %	DT2 vs. PT %
**Specific force (N/cm^2^)**									
TRA	20.9 ± 5.4	20.7 ± 4.4	20.7 ± 4.5	21.0 ± 4.7	2 ± 20	2 ± 19	3 ± 22	−1 ± 9	−1 ± 6
CON	22.3 ± 7.4	22.8 ± 7.6	23.0 ± 7.1	22.8 ± 7.8	2 ± 6	1 ± 5	2 ± 5	0 ± 3	2 ± 6
**KE MVC (N⋅m)**									
TRA	248 ± 688	275 ± 94^∗^	266 ± 92^∗^	259 ± 91^†^	13 ± 18^∗^	9 ± 19^∗^	6 ± 17	−4 ± 5	−6 ± 5^†^
CON	267 ± 76	259 ± 82	262 ± 71	268 ± 84	−1 ± 11	−1 ± 9	0 ± 14	2 ± 8	3 ± 13
**VL fascicle force (N)**									
TRA	1068 ± 365	1266 ± 347	1163 ± 339	1159 ± 313	–	–	–	–	–
CON	1723 ± 402	1698 ± 388	1768 ± 342	1788 ± 422					
**VL pennation angle (°)**									
TRA	16.0 ± 1.4	16.5 ± 1.3	16.2 ± 1.1	15.6 ± 1.4	4 ± 6^∗^	2 ± 8	−2 ± 8	−2 ± 4	−5 ± 4
CON	14.1 ± 6.3	14.0 ± 6.2	13.9 ± 6.2	14.0 ± 6.1	0 ± 4	−1 ± 5	0 4	−1 ± 2	0 ± 1
**VL fascicle length (mm)**									
TRA	101 ± 16	116 ± 16^∗^	112 ± 14^∗†^	106 ± 13^†^	16 ± 9^∗^	11 ± 9^∗^	6 ± 8	−4 ± 3^†^	−9 ± 5^†^
CON	108 ± 16	111 ± 15	112 ± 16	111 ± 17	3 ± 6	6 ± 6	3 ± 7	2 ± 3	−1 ± 3
**Sub fat (mm)**									
TRA	7.7 ± 6.2	6.9 ± 5.3	7.3 ± 5.8	7.7 ± 6.0	−9 ± 14	−1 ± 18	6 ± 26	10 ± 26	19 ± 40
CON	6.1 ± 4.7	6.4 ± 5.3	6.5 ± 5.3	6.4 ± 5.3	3 ± 12	8 ± 18	3 ± 12	5 ± 18	0 ± 1

*BL, baseline; PT, post-training; DT1, detraining 1; DT2, detraining 2; TRA, training group; CON, control group; KE MVC, knee extensor maximal voluntary contraction; Sub fat, subcutaneous fat; VL, vastus lateralis. ^∗^Significantly different compared to baseline, ^†^Significantly different to post-training.*

All Pθ data are presented in Table 1. There was a significant effect of time on absolute changes in Pθ, with the only significant difference found between Pθ at PT and DT2 in within TRA changes, with no significant difference between TRA and CON at any time point. There was a significant (*p* < 0.05) change in Pθ at PT compared to baseline and CON but not at DT1 or DT2 in TRA, with no changes (*p* > 0.05) in CON at any time point. There were no significant (*p* > 0.05) differences in Pθ at DT1 or DT2 compared to PT in TRA.

All L_F_ data are presented in Table 1. There were significant (*p* < 0.01) absolute increases in L_F_ at PT and DT1 compared to baseline and CON, but not at DT2 (*p* > 0.05) in TRA, with no changes in CON at any time point. There were also significant (*p* < 0.05) absolute decreases in L_F_ at DT1 and DT2 compared to PT. There were significant (*p* < 0.01) relative increases in L_F_ at PT and DT1 compared to baseline and CON, but not at DT2 (*p* > 0.05) in TRA, with no relative changes in CON at any time point. There were also significant (*p* < 0.05) relative decreases in L_F_ at DT1 and DT2 compared to PT.

There were no significant absolute or relative changes in SF compared to baseline at any time points (PT, DT1, and DT2) in TRA or CON (*p* > 0.05, Table 1). There were no correlations between subcutaneous fat levels and TNFα levels at any point.

### Knee Extensor Maximal Voluntary Contraction (KE MVC) and Vastus Lateralis Specific Force

**FIGURE 2:**
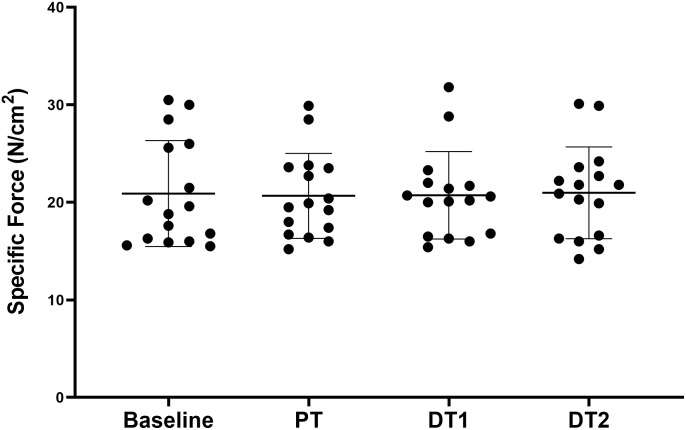
Changes in specific force in TRA group at baseline, post-training (PT), detraining 1 (DT1), and detraining 2 (DT2).

All torque data are presented in Table 1. There were significant absolute and relative increases (*p* < 0.05) in KE MVC at PT and DT1 compared to baseline in TRA with no changes in CON. There were no significant absolute decreases in KE MVC at DT1 (*p* > 0.05) although there were significant absolute decreases in KE MVC at DT2 (*p* < 0.01) compared to PT in TRA with no changes in CON. There was also a significant relative decrease in KE MVC at DT2 (*p* < 0.05) but not DT1 (*p* > 0.05) compared to PT in TRA with no changes in CON. All specific force data are presented in Table 1. There were no significant (*p* < 0.05) absolute or relative changes in specific force at any time point in TRA (Figure 2) or in CON.

### Tumor Necrosis Factor Alpha (TNFα)

**FIGURE 3:**
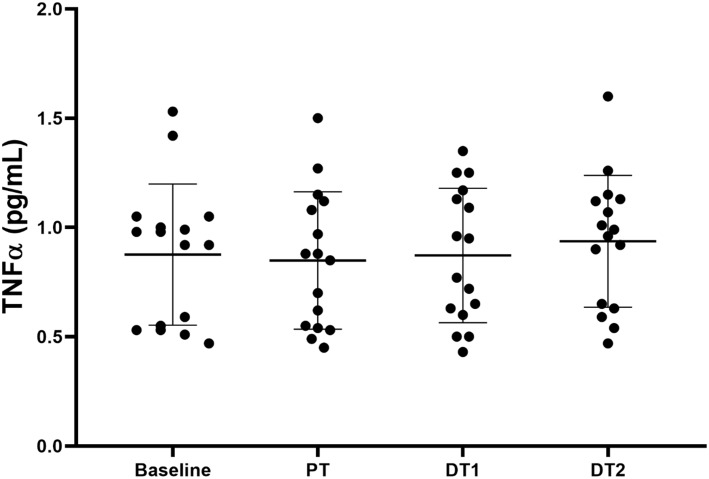
Changes in TNFα in TRA group at baseline, post-training (PT), detraining 1 (DT1), and detraining 2 (DT2).

There were no significant (*p* > 0.05) absolute or relative changes in TNFα compared to baseline or CON in TRA at any time point (Figure 3). There were also no significant absolute or relative changes in TNFα compared to PT at DT1 or DT2 (*p* > 0.05). However, there was a significant correlation (*p* = 0.022, *r* = 0.57) between the relative changes in TNFα and npCSA at DT2 compared to PT.

## Discussion

The main findings from the current study are that (1) following 8 weeks of RT there was no effect on systemic TNFα levels, and as such the training benefits were independent of this endocrine parameter, (2) changes in TNFα levels were significantly correlated with losses in muscle mass following 4 weeks of detraining, and (3) we report for the first time that a short period of DT does not appear to alter the quality (i.e., specific force) of the remaining skeletal muscle.

### Resistance Training and TNFα Levels

The role of physical activity is apparent, when taking into account its role in systemic inflammation. Indeed physical activity is believed to play a central role in the delicate balance between so-called myokines, which are cytokines produced by contracting skeletal muscles during physical activity. Examples of these myokines are interleukin (IL)-6 and TNF-α (Pratesi et al., 2013). Interestingly, IL-6 has always been considered a pro-inflammatory cytokine, but it might display anti-inflammatory effects during exercise (Pratesi et al., 2013). TNF-α on the other hand, can both stimulate muscle growth (Chen et al., 2007) but also lipid metabolism through minimizing the accumulation of lipids in adipocytes (Wang et al., 2013). Hence, reverting to a less-active lifestyle following training would cause an imbalance, resulting in a pro-inflammatory status and the beginning of a vicious cycle including enhancement of muscle loss, accumulation of intramuscular fat and a decrease in muscle strength. This is more or less confirmed by previous studies showing that low physical activity and/or sedentary behavior is indeed associated with skeletal muscle atrophy (Degens and Alway, 2006; Bostock et al., 2013). On the contrary, physical activity can provide a physiological stimulus, making the skeletal muscle behave as an endocrine organ, counteracting inflamed aging (Pratesi et al., 2013). It is also interesting in the current study that pooled baseline TNFα levels tended to be correlated (*p* = 0.055, *r* = 0.41) with baseline npCSA in the young population. This would also reflect the evidence showing that TNFα has a dose-dependent relationship on MHC loss in skeletal muscle (Li et al., 1998). It is well established that a chronic low-grade inflammatory profile (CLIP) is associated with TNFα levels and age-related Sarcopenia in older populations (Beyer et al., 2012). Our current data suggests that there may be a possible role of pro-inflammatory cytokine modulation of muscle mass even in the younger, healthy adult. However, this is purely speculative at this point.

Previous research using young participants found that the mRNA of TNFα was significantly increased immediately and remained elevated up to 24 h post-resistance exercise (Louis et al., 2007), which is in contrast to the findings of Franchi et al. (2014) who did not detect changes in TNFα phosphorylation after concentric or eccentric resistance exercise. However, circulating TNFα levels appear to display a shorter temporal pattern. Townsend et al. (2013) showed with the acute response to RT, circulating TNFα was significantly increased immediately post-RT compared to pre-RT in resistance trained men. However, TNFα levels had returned to baseline within 30 min post-RT, remaining as such for a further 24 and 48 h. Similarly, Alves et al. (2013) demonstrated that following either eccentric or concentric RT only, there were no acute increases in TNFα post-RT (24–96 h). In addition, Peake et al. (2006) reported following submaximal and maximal lengthening contractions, no significant changes in circulating TNFα in either acute windows of 1–3 h or 1–4 days. The results from the current study show that systemic levels of TNFα are not significantly altered by an 8-week RT program. This is also in somewhat of agreement with the study of Alves et al. (2013) whom reported that following 10 RT sessions over 3 weeks, there were no significant changes in circulating TNFα 24–96 h post the final RT session. The current study is slightly in contrast with that of Paoli et al. (2015). These authors report that following 8 weeks of upper body RT in combination with either a high-or-low protein diet, chronic RT could elevate both the basal and RT-induced response of circulating TNFα. The pre-, post-RT baseline changes in circulating TNFα levels (5.9 ± 0.46 ng/L to 15.79 ± 7.4 ng/L in normal protein diet, and 6.44 ± 0.65 ng/L to 11.04 ± 2.16 ng/L in high protein diet) were not directly compared statistically, rather the authors compared differences in the acute response to RT at baseline and post 8 weeks RT. Therefore it is still unclear to the exact effects of RT on chronic circulating TNFα levels. In addition the authors of the aforementioned study, however, did not report either changes in muscle mass or strength, which further compounds the lack of clarity of what effects marginal changes in TNFα levels may have, if any at all. Using a slightly different approach, Rall et al. (1996b) used peripheral blood mononuclear cells (PBMC) subpopulations under various conditions to investigate the inflammatory response of a young adult population versus rheumatoid arthritis and healthy elderly populations to 12 weeks of progressive RT. There were no changes in TNFα production in PBMC under any condition in the young healthy adults following RT. It is worth noting that despite this, the changes in young adults’ strength was significantly increased post-RT with data shown in a previous study (Rall et al., 1996a). This is in agreement with the observations of the current study with participants undergoing significant improvements in strength with no changes in TNFα. In a study by Ihalainen et al. (2018), the authors found that systemic TNFα were significantly reduced following 24 weeks of combined aerobic and RT in young men. However, due to 3-fold greater duration of training, lack of particular details around the RT program variables and the use of males only, make any comparisons between the current study and that of Ihalainen et al. (2018) very difficult. Furthermore, the acute response of TNFα to RT exercise and endurance exercise have been found to be disparate (Louis et al., 2007). These observations therefore again highlight the need for concurrent aerobic and RT programs to be treated independently from RT programs only, and that a combination of training modes (RT + aerobic training) may be more beneficial for modulating pro-inflammatory cytokines. The lack of change in TNFα levels following a RT only program are consistent with those found in previous research in a different age-based population. In that particular study, 11 middle-aged sedentary men (49 ± 5 years) completed 16 weeks of RT with no change in TNFα levels (Libardi et al., 2012). However, it is worth noting the baseline and post-RT TNFα levels were 3-fold greater than the current study and could be verging on clinical levels (Milani et al., 1996). In older populations TNFα levels have been shown to be sensitive to a RT program with reductions in TNFα levels reported following the completion of such programs (Greiwe et al., 2001; Tomeleri et al., 2016) with baseline TNFα receptor levels inversely correlated with changes in strength (Bruunsgaard et al., 2004). Reductions in systemic TNFα following RT in older populations it must be pointed out are not universally found, with some studies also showing no alterations to TNFα levels (Onambélé-Pearson et al., 2010a,b). Therefore in contrast to the above data regarding sensitivity of TNFα to RT in older populations, it appears that following a RT program in young, healthy participants, systemic TNFα is not sensitive to RT-induced changes in muscle size and/ or function.

Interestingly, our data demonstrates that the relative decrease in muscle npCSA from post-RT to 4 weeks of detraining was significantly correlated with the change in TNFα levels over the same period, which tended to increase. Acute systemic administration of TNFα has been shown to increase the expression of ubiquitin mRNAs in soleus muscles (Llovera et al., 1997) and reduce protein synthesis rates of myofibrillar and sarcoplasmic proteins in the gastrocnemius (Lang et al., 2002). However, *in vitro* work has demonstrated that superimposition of anabolic stimuli can ablate the effects of TNFα on muscle protein loss (Guttridge et al., 2000). Therefore during a period of detraining, where there is a lack of anabolic stimuli, changes in TNFα levels may mediate the loss of muscle mass, however, further work is needed to support this. Previous work on TNFα suggests these effects are modulated through TNFα’s upstream effects on the p38 MAPK and nuclear factor-κB (NF- κB) pathways of the ubiquitin-proteasome pathway (Li et al., 1998, 2005; Li and Reid, 2000).

### Specific Force

Vastus lateralis muscle specific force did not change in the current study following RT. This is in contrast to the only other published study that has investigated *in vivo* muscle specific force changes following RT in young individuals. Erskine et al. (2010) demonstrated that following 9 weeks of RT, VL muscle specific force increased by 20% in 17 untrained males. The methods and RT used by Erskine et al. (2010) were very similar to those of the current study, however, importantly in the current study we did not assess muscle activation levels using the interpolated twitch technique. Despite this there is currently no consensus as to whether maximal muscle activation, whether supramaximally stimulated or not, is actually increased following a RT program (Folland and Williams, 2007). This therefore means that the current data can still be accurately representative of muscle specific force changes following RT. Additionally, the study of Erskine et al. (2010) used males only, however, in the current study 50% of the training group were females. No data is currently available on whether there are sex differences in muscle specific force, although we have previously demonstrated that males and females have similar relative muscle adaptations to a RT program, and the authors do not think this explains the differences between these studies. A reason why there were no observed changes in muscle specific force in the current study is that both the npCSA (muscle size) and torque (muscle strength) were both significantly enhanced following training, but none more so than the other. In other studies, muscle strength has increased to a larger degree than muscle size [e.g., Erskine et al. (2010)] however, due to the heterogeneous responses of individuals to changes in muscle size and strength, this relationship was not apparent in the current study. In conjunction with this, it should also be pointed out there were no correlations between changes in muscle size and strength following RT and during DT in the present study.

Changes in npCSA have not been reported previously following a short-term period of detraining such as that used in the current study, nor has specific force been reported during any detraining literature. Many human muscles, including the quadriceps are pennate muscles, and therefore aCSA measurements underestimate pCSA (Wickiewicz et al., 1983). The pCSA represents the area of sarcomeres arranged in parallel and thus the maximum amount of cross-bridges that can be formed during a contraction and is the primary determinant of maximal muscle force. Importantly, we also normalized the measured pCSA to body mass through allometric scaling which is an influential factor when comparing individuals with heterogeneous body composition. There were significant reductions in npCSA following both two (−6 ± 8%) and 4 weeks (−10 ± 8%) of DT compared to PT (mean −2.5% per week). This short term reduction in muscle mass is consistent with previous observations that used anatomical CSA measurements (McMahon et al., 2012; Yasuda et al., 2015). However, as both of these previous studies investigated the effects of two different training regimens on muscle size, effects of training and detraining were not the same between these groups. Despite the aforementioned loss of muscle mass, npCSA of the VL muscle was still significantly increased relative to baseline after 4 weeks of detraining. Again this is in agreement with the data of Yasuda et al. (2015) that showed following 6 weeks of RT and a subsequent 3 weeks of DT, pectoral and triceps brachii CSA was still improved compared to baseline. Trends for changes in strength mirrored those of npCSA in the main, with significant reductions in strength at DT1 and DT2 compared to PT. KE MVC remained greater compared to baseline at DT1, however, this had diminished by DT2. Previous data has shown that muscle strength was maintained following 1 month and up to 3 months of detraining following 3 months of RT (Kubo et al., 2010). The previous authors suggested this was in part due to neural adaptations. This may explain in part the differences observed between the studies. In the current study, our participants completed 8 weeks of RT using dynamic muscle contractions and were assed for maximal voluntary contraction (MVC) via isometric contractions. Kubo et al. (2010) participants were trained and assessed using isometric contractions with a 50% longer training duration. Therefore the neural specificity of adaptations to training and testing protocol, and longer training time may explain some of the differences (Folland and Williams, 2007). This is the first study to report muscle specific force during a period of DT of any length. Muscle specific force accounts for many of the other extrinsic factors present in analyzing the relationship between muscle size and strength such as angle of fiber insertion to the aponeurosis, antagonist co-contraction, moment arm length etc., and requires specific measurements such as force at optimum fiber length, pCSA, muscle architecture and muscle volume for example. Specific force has been found to range between 10 and 40 N.cm^−2^ in human single muscle fibers *in situ* [e.g., Degens et al. (1999)] and between 10 and 100 N.cm^−2^ in humans *in vivo* (Narici et al., 1988; Maganaris et al., 2001). The muscle specific force ranged from 14.2 to 30.5 N.cm^−2^ in the current study which is also similar to that previously reported in the VL muscle *in vivo* in humans (Maganaris et al., 2001; Reeves et al., 2004b; Erskine et al., 2010). Muscle specific force did not change during DT, as mean VL fascicle force declined by −8% from PT to DT2 which was almost identical (−10%) to the observed mean reduction in npCSA during the same time period. Therefore, the data from the current study suggests that the intrinsic force generating capabilities of the muscles do not change following short term detraining.

### Limitations

There are two main limitations in the current study that are important to highlight. First, the estimation of pCSA is derived from an estimate of muscle volume using three axial aCSA measures at 25, 50, and 75% of femur length. The aCSA estimates are themselves made up of composite axial ultrasound scans. Therefore there is the potential to over or underestimate VL muscle volume, and thus pCSA. However, aCSA measures using the technique outlined in the current study have been found to be in almost perfect agreement with MRI measures, with a very small typical error of only 1.7% between the two estimates along the length of the VL muscle (Reeves et al., 2004a). Furthermore, when multiple ultrasound axial aCSA scans are combined to estimate VL muscle volume, there are no significant differences between the derived muscle volumes of ultrasound and MRI, with a 2.2% mean difference between estimates, which was deemed not clinically relevant (Walton et al., 1997). Furthermore, Morse et al. (2007) demonstrated that just a single aCSA axial scan of the quadriceps at either 40, 50, or 60% of femur length correlates significantly and highly (*R* = 0.84, 0.93, and 0.90, respectively) with the observed quadriceps volume as measured by MRI. In fact, a single scan at 50 or 60% femur length is associated with standard error of the estimate (SEE) of only 13 and 10%, respectively. As such, we would argue that taking three axial scans along the femur length leads to an estimated VL volume with sufficient accuracy, particularly as the pCSA calculated that include such error estimates fall comfortably within the relative changes in pCSA in our results. Lastly, Morse et al. (2007) reported a mean ± S.D. VL muscle volume of 702 ± 108 cm^3^ in a group of 18 young recreationally active men. The mean ± S.D. estimated VL volume at baseline of the current study’s males within the training group (*n* = 8) was 741 ± 102 cm^3^, and therefore indicates that our current baseline measure of muscle volume are comparable to that of a similar homogenous population. The second limitation in the assessment of muscle architecture with particular reference to fascicle length, was the use of a 4 cm probe as the entire muscle fascicle cannot be recorded in one image. Furthermore, due to the 2D image, the fascicle length may be underestimated when the digitized fascicles do not lie in the image plane. Therefore, future studies investigating this may employ a probe with a scanning width of 6 or 10 cm, or even dual probe approach due to the relative length of VL fascicles (Brennan et al., 2017; Franchi et al., 2018). Finally, a further limitation was the use of a notch filter in the EMG recording system, to remove mains hum, which would have removed the physiological signal at this frequency.

## Conclusion

Following a prolonged period of RT in young healthy individuals, muscle mass and strength were increased although there were no effects on the specific force of muscle or on systemic levels of TNFα. Furthermore, following a period of short-term detraining, muscle mass and strength were significantly reduced with muscle mass remaining significantly above baseline values following the conclusion of the detraining period. Changes in TNFα were significantly correlated with the reductions in muscle mass following detraining compared to post-training. TNFα may play a part in the detraining induced loss of muscle mass following a period of RT. However, further work is needed to corroborate this and the mechanisms by which this may occur are currently unknown.

## Ethics Statement

This study was carried out in accordance with the recommendations of Manchester Metropolitan University Cheshire Campus ethics committee with written informed consent from all subjects. All subjects gave written informed consent in accordance with the Declaration of Helsinki. The protocol was approved by the Manchester Metropolitan University Cheshire Campus ethics committee.

## Author Contributions

GM, CM, KW, AB, and GO conceived and planned the experiments, supervised the training sessions and contributed to the final version of the manuscript. GM with the support of GO performed all the experiments, statistical analyses, and wrote the manuscript.

## Conflict of Interest Statement

The authors declare that the research was conducted in the absence of any commercial or financial relationships that could be construed as a potential conflict of interest.
